# Supplementary material for: Transkingdom interactions between Lactobacilli and hepatic mitochondria attenuate western diet-induced diabetes
Source: Nat Commun. 2021 Jan 4;12:101. doi: 10.1038/s41467-020-20313-x (PMC7782853; doi:10.1038/s41467-020-20313-x)
Supplement: Supplementary file 1 — Supplementary Information [file 41467_2020_20313_MOESM1_ESM.pdf]

Supplementary Figures

Transkingdom interactions between *Lactobacilli* and hepatic mitochondria attenuate western diet induced diabetes

Richard R. Rodrigues1#, Manoj Gurung2#, Zhipeng Li2#, Manuel García-Jaramillo3, Renee Greer2, Christopher Gaulke4, Franziska Bauchinger5, Hyekyoung You2, Jacob W. Pederson2, Stephany Vasquez-Perez2, Kimberly D. White2, Briana Frink2, Benjamin Philmus1, Donald B. Jump3, Giorgio Trinchieri6, David Berry5, Thomas J. Sharpton4, Amiran Dzutsev6, Andrey Morgun1*, Natalia Shulzhenko2*

[1]College of Pharmacy, [2]Veterinary Medicine, [3]College of Public Health and Human Sciences and [4]College of Science, Oregon State University, Corvallis, OR, USA; [5]Department of Microbiology and Ecosystem Science, University of Vienna, Vienna, Austria; [6]Cancer and Inflammation Program, Center for Cancer Research, National Cancer Institute, National Institutes of Health, Bethesda, MD, USA

#equal contribution

*co-senior authors

Correspondence to: andriy.morgun@oregonstate.edu or natalia.shulzhenko@oregonstate.edu

Supplementary Fig 1

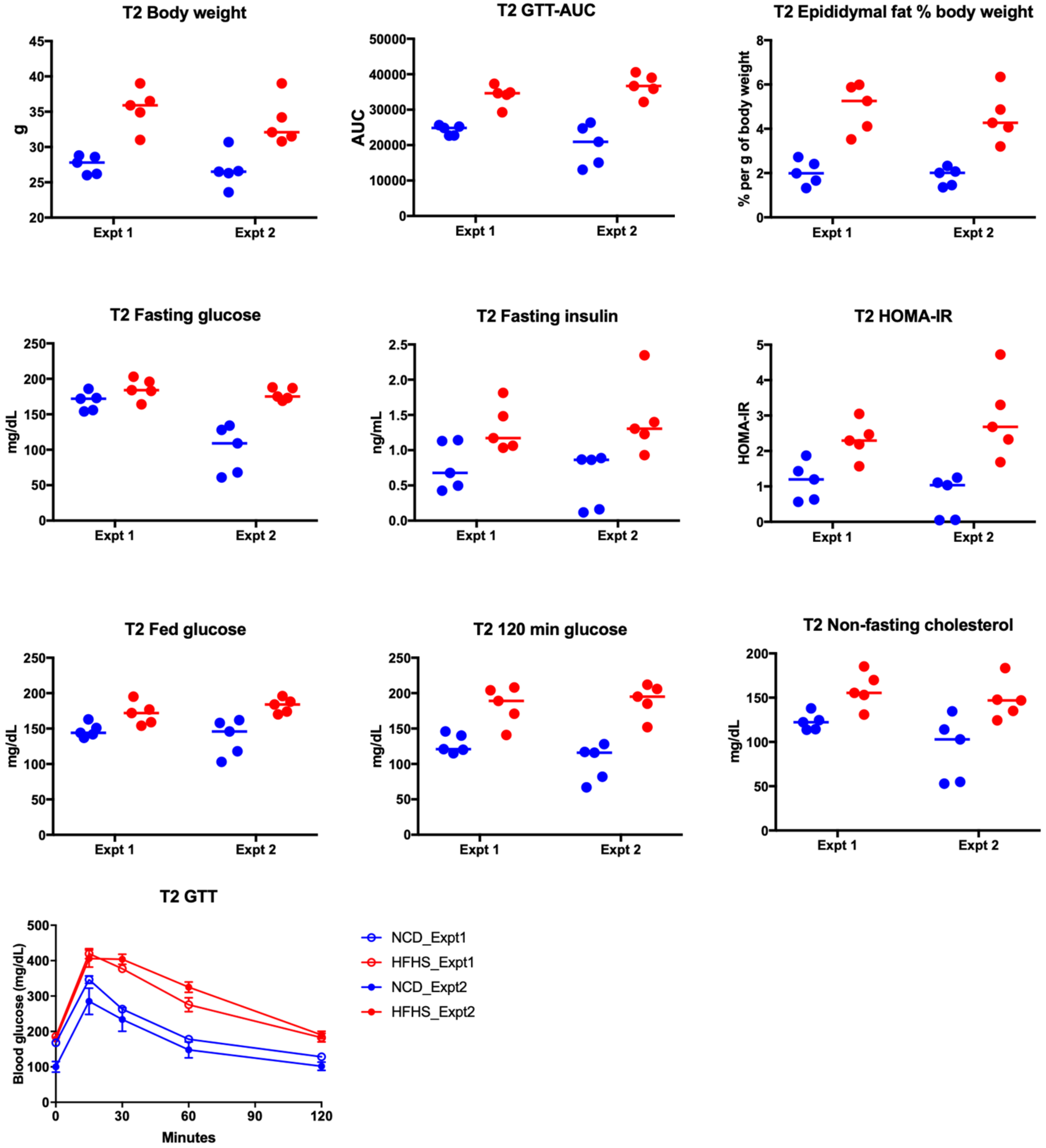

Figure S1: Changes in metabolic parameters due to diet.
The blue and red colors indicate levels of metabolic parameters measured in mice fed normal diet (ND, blue) or western diet (WD, red) for 8 weeks. All these parameters are statistically significant (two-tail Mann-Whitney p-value < 20% in each experiment, Fisher's p-value over experiments < 5%, and FDR < 10%). Each symbol represent mouse, lines are median values. Source data are provided as a Source Data file.

Supplementary Fig 2

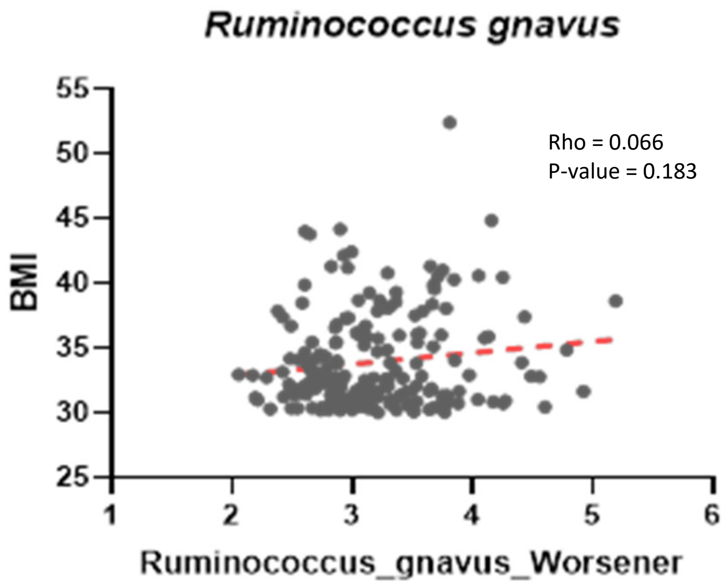

Figure S2: Spearman correlation of *Ruminococcus gnavus* abundance in stool with BMI of obese human. The Spearman rho correlation co-efficient and one-tail p-value is provided.

Supplementary Fig 3

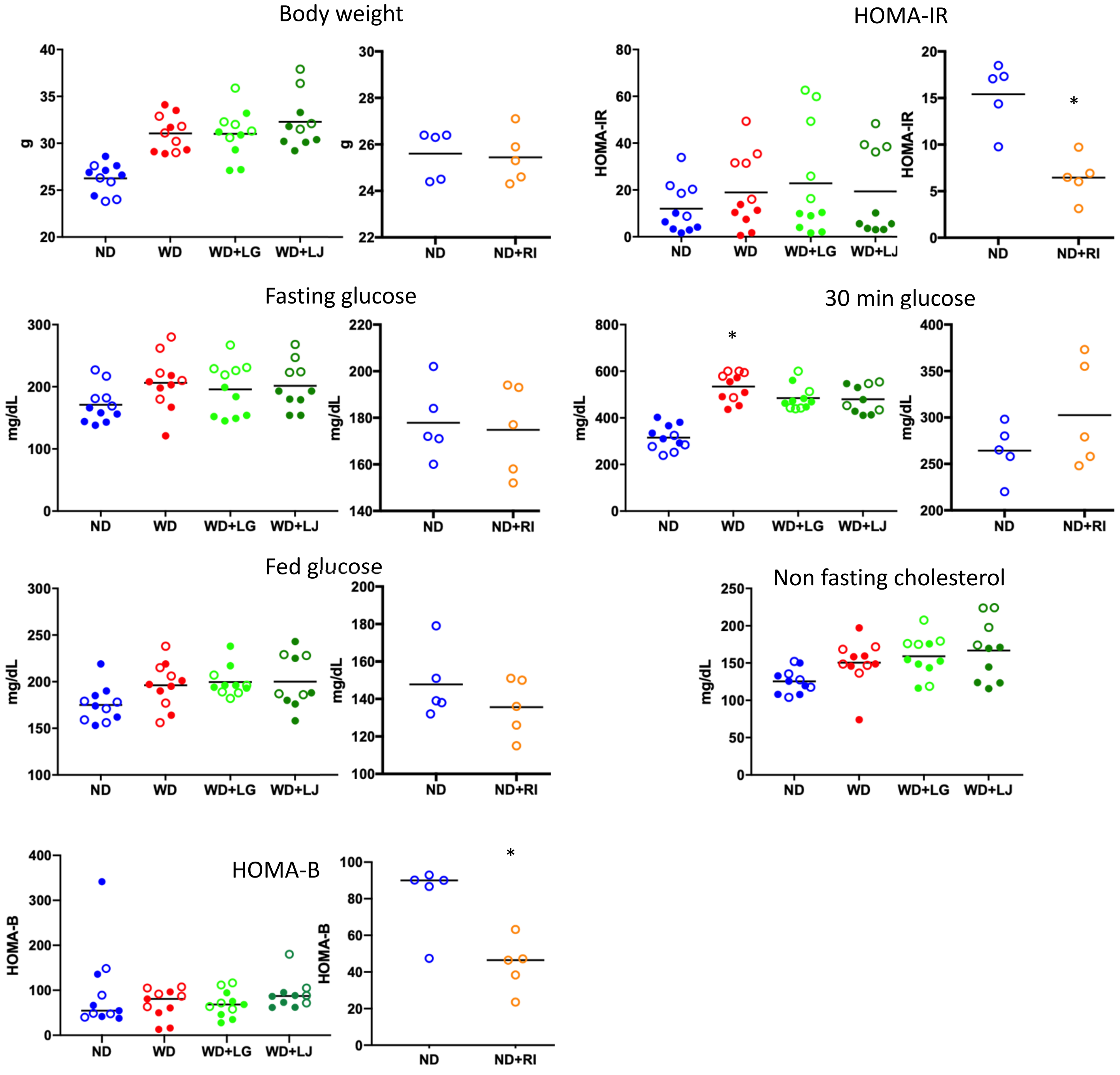

Figure S3: Changes in metabolic parameters due to supplementation of candidate microbes. The blue, red, light green, dark green, and orange colors indicate ND, WD, WD with *L. gasseri*(WD+LG), WD with *L. johnsonii* (WD+LJ), ND with R. ilealis (ND+RI), respectively. The metabolic parameters in mice supplemented with or without candidate microbe. Open and closed circles indicate 2 independent experiments. * indicates statistically significant differences in levels of the parameter between control group (WD for *Lactobacilli*, ND for *R. ilealis*) versus those supplemented with bacteria (one-tail t-test p-value < 5% with FDR <15%. Each symbol represents mouse, lines are median values, all replicates are shown. Source data are provided as a Source Data file.

Supplementary Figure 4

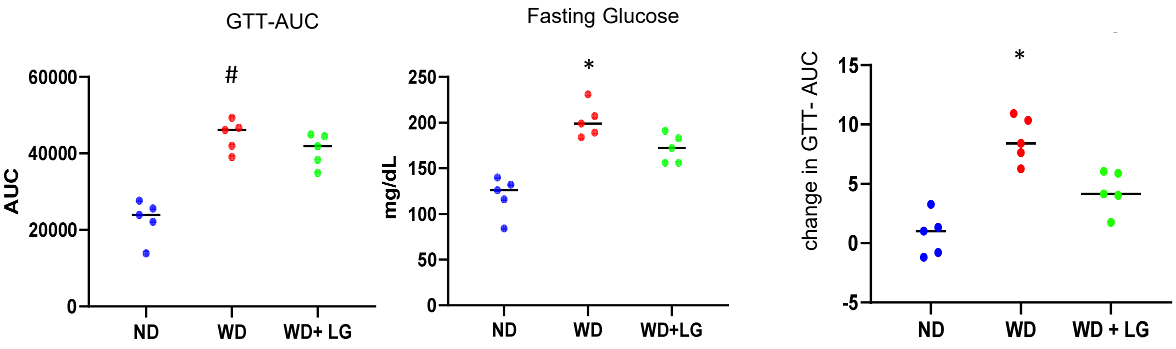

Figure S4: Glucose tolerance test and fasting glucose in mice supplemented with L. gasseri (LG) after 8 weeks on WD. The right figure shows the change in glucose tolerance test (AUC) after 12 weeks of WD (which was 4 wks of LG), calculated by subtracting the 12 week AUC of each mouse from 8 week AUC (when mice were on WD only) and normalizing by median of AUC change of ND group. * indicates significant change between control group (WD and WD + LG, one-sided p-value < 5%, Mann-Whitney Test, median). # indicates p-value of 0.07. Each symbol represent mouse, lines are median values, all replicates are shown. Source data are provided as a Source Data file.

Supplementary Fig 5

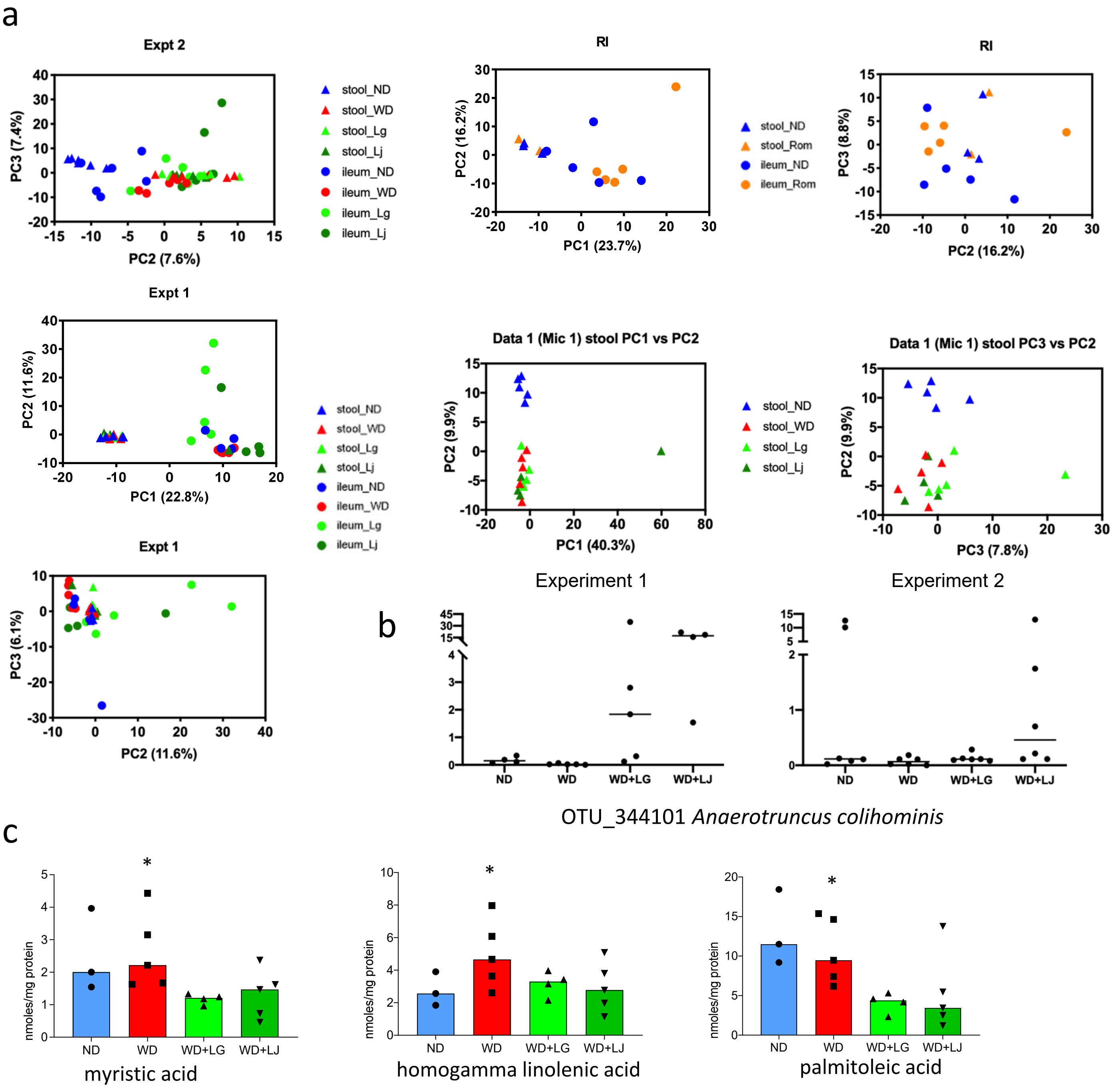

Figure S5: Effect of microbial supplementation on microbial communities and fatty acids.

a) Principal Component Analysis of stool (triangle) and ileal (circle) microbial communities of mice on ND, WD, WD+LG, WD+LJ, or RI. b) The ileal abundance of *Anaerotruncus colihominis* in ND, WD, WD+LG, and WD+LJ fed mice. c) The levels of long chain fatty acids in liver of mice supplemented with or without *Lactobacillus* candidates. Asterisk indicates statistically significant differences between WD and groups supplemented with bacteria (one-sided t-test p-value < 5%, except for # p<8%). Each dot represents a mouse, all replicates are shown. Source data are provided as a Source Data file.

Supplementary Figure 6

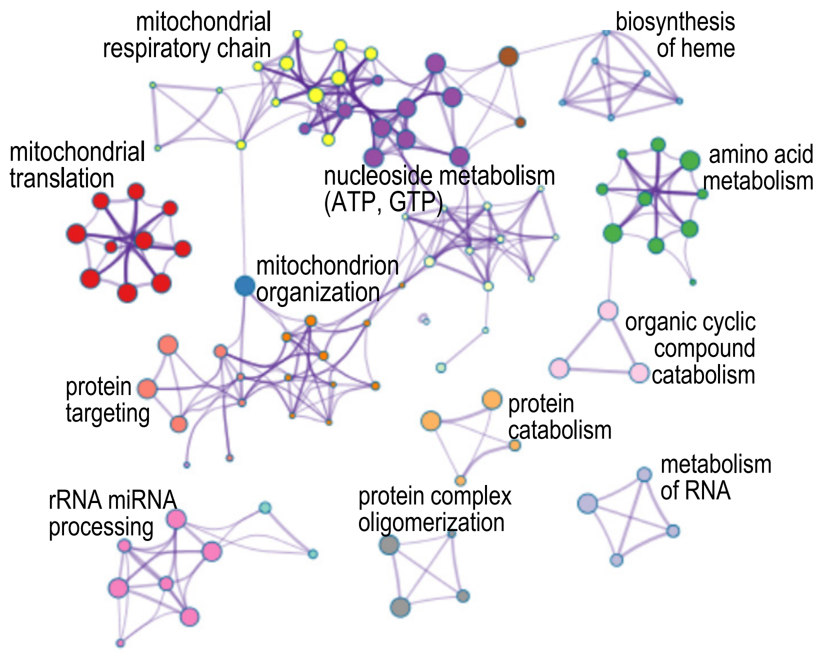

Figure S6: Functional enrichment for the liver genes upregulated by the *Lactobacillus gasseri/johnsonii*. Pathways identified by Metascape (http://metascape.org).

Supplementary Figure 7

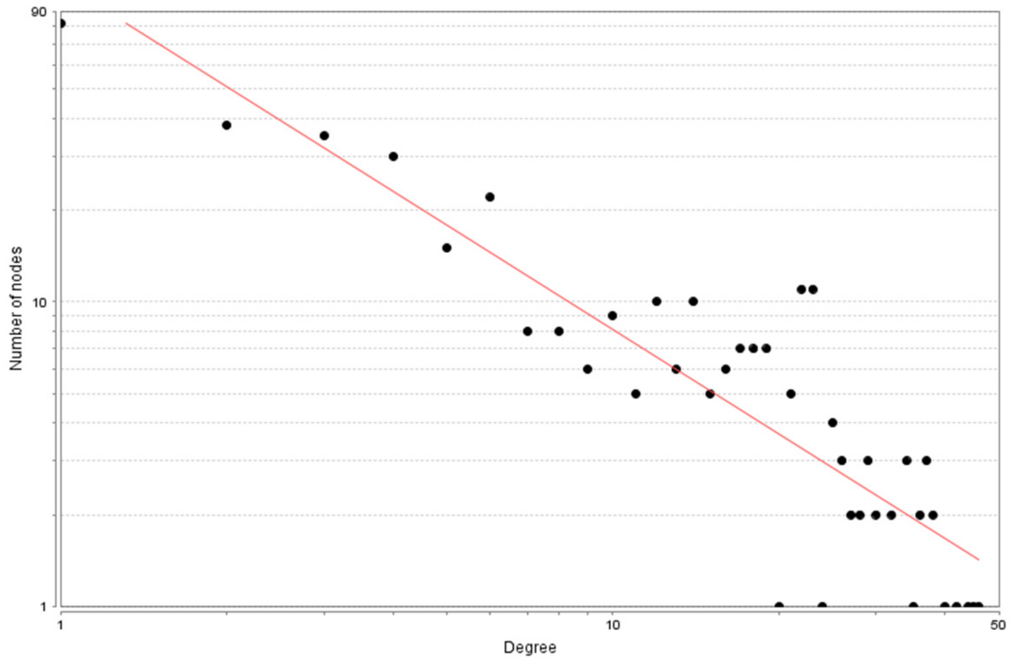

Figure S7: Power law distribution of nodes in multi-omics network
