# Supplementary material for: Transkingdom interactions between Lactobacilli and hepatic mitochondria attenuate western diet-induced diabetes
Source: Nat Commun. 2021 Jan 4;12:101. doi: 10.1038/s41467-020-20313-x (PMC7782853; doi:10.1038/s41467-020-20313-x)
Supplement: Supplementary file 3 — Description of Additional Supplementary Files [file 41467_2020_20313_MOESM3_ESM.pdf]

Description of Additional Supplementary Files

File Name: Supplementary Data1-16-oct29v2.xlsx

Description: Additional results derived from the various analyses described in the accompanying manuscript.
