# Supplementary material for: Transkingdom interactions between Lactobacilli and hepatic mitochondria attenuate western diet-induced diabetes
Source: Nat Commun. 2021 Jan 4;12:101. doi: 10.1038/s41467-020-20313-x (PMC7782853; doi:10.1038/s41467-020-20313-x)
Supplement: Supplementary file 5 — Reporting Summary [file 41467_2020_20313_MOESM5_ESM.pdf]

nature research

Corresponding author(s): Natalia Shulzhenko/Andrey Morgun

Last updated by author(s): Oct 9, 2019

Reporting Summary

Nature Research wishes to improve the reproducibility of the work that we publish. This form provides structure for consistency and transparency in reporting. For further information on Nature Research policies, see Authors & Referees and the Editorial Policy Checklist.

Statistics

For all statistical analyses, confirm that the following items are present in the figure legend, table legend, main text, or Methods section.

n/a	Confirmed	
☐	☒	The exact sample size (n) for each experimental group/condition, given as a discrete number and unit of measurement
☐	☒	A statement on whether measurements were taken from distinct samples or whether the same sample was measured repeatedly
☐	☒	The statistical test(s) used AND whether they are one- or two-sided<br>*Only common tests should be described solely by name; describe more complex techniques in the Methods section.*
☐	☒	A description of all covariates tested
☐	☒	A description of any assumptions or corrections, such as tests of normality and adjustment for multiple comparisons
☐	☒	A full description of the statistical parameters including central tendency (e.g. means) or other basic estimates (e.g. regression coefficient) AND variation (e.g. standard deviation) or associated estimates of uncertainty (e.g. confidence intervals)
☐	☒	For null hypothesis testing, the test statistic (e.g. F, t, r) with confidence intervals, effect sizes, degrees of freedom and P value noted<br>*Give P values as exact values whenever suitable.*
☒	☐	For Bayesian analysis, information on the choice of priors and Markov chain Monte Carlo settings
☒	☐	For hierarchical and complex designs, identification of the appropriate level for tests and full reporting of outcomes
☒	☐	Estimates of effect sizes (e.g. Cohen's d, Pearson's r), indicating how they were calculated

Our web collection on statistics for biologists contains articles on many of the points above.

Software and code

Policy information about availability of computer code

Data collection	No special software was used for data collection.
Data analysis	Data was analyzed using QIIME (v1), GraphPad Prism (v7 & v8.4.1), FIJI (i.e. ImageJ, v2.0.0-rc-69/1.52i), R packages: seqtime 0.1.1; igraph 1.2.5 Progenesis QI software (Version 2.3). Custom codes available at https://github.com/richrr/TransNetDemo, https://github.com/fbauchinger/keystone_species_model

For manuscripts utilizing custom algorithms or software that are central to the research but not yet described in published literature, software must be made available to editors/reviewers. We strongly encourage code deposition in a community repository (e.g. GitHub). See the Nature Research guidelines for submitting code & software for further information.

Data

Policy information about availability of data

All manuscripts must include a data availability statement. This statement should provide the following information, where applicable:

- Accession codes, unique identifiers, or web links for publicly available datasets
- A list of figures that have associated raw data
- A description of any restrictions on data availability

The datasets generated during and/or analysed during the current study are available at SRA (PRJNA558801), GEO (GSE136033, Metabolomics Workbench under ST001436; supplementary tables, and ndexbio with hyperlinks provided. The Transmission Electron Microscopy image or any other associated data is available from the corresponding author upon reasonable request.

Field-specific reporting

Please select the one below that is the best fit for your research. If you are not sure, read the appropriate sections before making your selection.

☒ Life sciences ☐ Behavioural & social sciences ☐ Ecological, evolutionary & environmental sciences

Life sciences study design

All studies must disclose on these points even when the disclosure is negative.

Sample size	Sample size calculation was not performed. The number of samples was decided based on previously published research (Rodrigues et al., 2017, Front Microbiol. 2017 Nov 22;8:2306. doi: 10.3389/fmicb.2017.02306)
Data exclusions	One datapoint was excluded because it was an outlier as per the (default) ROUT method in GraphPad Prism (v8). This one datapoint was from Fig. 5f where one value of one concentration of Glutahione treatment group was removed.
Replication	The mice of same gender and age were ordered from the same room and facility (Jackson) and protocols were adhered to. Experiments were repeated and reproducible. Experiments were independently repeated at least twice for all but the one of suppmentation with R. ilealis
Randomization	The mice and cell culture wells were randomly allocated into experimental groups.
Blinding	The investigators were not blinded to group allocation except for Transmission Electron Microscopy image analysis where first pairs of damaged (bright, lucent) and healthy mitochondria (dark, dense) were identified in each image. Next, quantitative data was extracted for several image parameters and analyzed to identify which of those differed between damaged and healthy mitochondria. The identification and selection have been performed “blindly” (i.e. the image analyst was unaware of the experimental group of samples). Blinding for other experiments was not possible because those who were doing the experiments also analyzed the data and plotted them.

Reporting for specific materials, systems and methods

We require information from authors about some types of materials, experimental systems and methods used in many studies. Here, indicate whether each material, system or method listed is relevant to your study. If you are not sure if a list item applies to your research, read the appropriate section before selecting a response.

Materials & experimental systems

n/a	Involved in the study	
☒	☐	Antibodies
☐	☒	Eukaryotic cell lines
☒	☐	Palaeontology
☐	☒	Animals and other organisms
☒	☐	Human research participants
☒	☐	Clinical data

Methods

n/a	Involved in the study	
☒	☐	ChIP-seq
☒	☐	Flow cytometry
☒	☐	MRI-based neuroimaging

Eukaryotic cell lines

Policy information about cell lines

Cell line source(s)	AML12 (ATCC® CRL-2254™) cells line was obtained from Dr. Donald Jump's lab at the Oregon State University
Authentication	no authentication
Mycoplasma contamination	not tested
Commonly misidentified lines (See ICLAC register)	No commonly misidentified cell lines were used.

Animals and other organisms

Policy information about studies involving animals; ARRIVE guidelines recommended for reporting animal research

Laboratory animals	Seven weeks old, C57BL/6 male mice were purchased from Jackson Laboratories (Bar Harbor, Maine). Mice were housed at the Laboratory Animal Resource Center at the Oregon State University under standard 12-h light cycle and an ambient temperature of 22±1 °C and humidity around 45%.
Wild animals	The study did not involve wild animals.
Field-collected samples	The study did not involve samples collected from the field.
Ethics oversight	Oregon State University Institutional Animal Care and Use Committee approved this study protocol

Note that full information on the approval of the study protocol must also be provided in the manuscript.
